# Supplementary material for: Metabolic Pathway Analysis of Nitrogen and Phosphorus Uptake by the Consortium between C. vulgaris and P. aeruginosa
Source: Int J Mol Sci. 2019 Apr 23;20(8):1978. doi: 10.3390/ijms20081978 (PMC6515159; doi:10.3390/ijms20081978)
Supplement: Supplementary file 1 [file ijms-20-01978-s001.zip › ijms-464067 supplementary/Table S1.pdf]

Reaction number	Metabolism	E.C. number
1	Photosynthesis	PS1-PSII
2	Photosynthesis	N/A
3	Photosynthesis	3.6.3.14
4	External exchange	
5	External exchange	
6	Calvin cycle	4.2.1.1
7	Calvin cycle	4.1.1.39
8	Calvin cycle	2.7.2.3
9	Calvin cycle	1.2.1.13
10	Internal exchange	
11	Calvin cycle	5.3.1.1
12	Calvin cycle	4.1.2.13
13	Calvin cycle	3.1.3.11
14	Calvin cycle	5.3.1.9
15	Calvin cycle	2.2.1.1
16	Calvin cycle	4.1.2.13
17	Calvin cycle	3.1.3.37
18	Calvin cycle	2.2.1.1
19	Calvin cycle	5.1.3.1
20	Calvin cycle	5.3.1.6
21	Calvin cycle	2.7.1.19
22	External exchange	
23	Glucolysis	2.7.1.1
24	Glucolysis	5.3.1.9
25	Glucolysis	2.7.1.11
26	Glucolysis	3.1.3.11
27	Glucolysis	4.1.2.13
28	Glucolysis	5.3.1.1
29	Glucolysis	1.2.1.12
30	Glucolysis	2.7.2.3
31	Glucolysis	1.2.1.9
32	Glucolysis	5.4.2.12
33	Glucolysis	4.2.1.11
34	Glucolysis	2.7.1.40

35	Glucolysis	1.2.4.1
36	Glucolysis	4.1.1.1
37	Glucolysis	1.2.1.3
38	Glucolysis	6.2.1.1
39	Krebs cycle	6.4.1.1
40	Krebs cycle	2.3.3.1
41	Krebs cycle	4.2.1.3
42	Krebs cycle	4.2.1.3
43	Krebs cycle	1.1.1.41
44	Krebs cycle	1.2.4.2
45	Krebs cycle	1.2.4.2
46	Krebs cycle	2.3.1.61
47	Krebs cycle	1.8.1.4
48	Krebs cycle	6.2.1.5
49	Krebs cycle	1.3.5.1
50	Krebs cycle	4.2.1.2
51	Krebs cycle	1.1.1.37
52	Krebs cycle	4.1.1.49
53	Pentose Phosphate	1.1.1.49
54	Pentose Phosphate	3.1.1.31
55	Pentose Phosphate	1.1.1.44
56	Pentose Phosphate	5.3.1.6
57	Pentose Phosphate	5.1.3.1
58	Pentose Phosphate	2.2.1.1
59	Pentose Phosphate	2.2.1.1
60	Pentose Phosphate	2.2.1.2
61	Pentose Phosphate	2.7.6.1
62	Fatty acid biosynthesis	
63	Fatty acid biosynthesis	
64	Fatty acid biosynthesis	
65	Fatty acid biosynthesis	
66	Fatty acid biosynthesis	
67	Fatty acid biosynthesis	
68	Fatty acid biosynthesis	
69	Fatty acid biosynthesis	
70	Fatty acid biosynthesis	
71	Fatty acid biosynthesis	
72	Fatty acid biosynthesis	2.3.1.20

73	Oxidative phosphorylation	
74	Oxidative phosphorylation	3.6.3.14
75	Assimilation of Nitrogen	NR, 1.7.7.1
76	Assimilation of Nitrogen	1.4.1.4
77	Assimilation of Nitrogen	6.3.1.2
78	Asimilación de Nitrógeno	1.4.1.14
79	Amino acid biosynthesis: Alanine	2.6.1.2
80	Amino acid biosynthesis: Aspartate	2.6.1.1
81	Amino acid biosynthesis: Serine	1.1.1.95
82	Amino acid biosynthesis: Serine	2.6.1.52
83	Amino acid biosynthesis: Serine	3.1.3.3
84	Amino acid biosynthesis: Glycine	
85	Amino acid biosynthesis: Homoserin	1.1.1.3
86	Amino acid biosynthesis: Homoserin	2.7.1.39
87	Amino acid biosynthesis: Homoserin	4.2.3.1
88	Amino acid biosynthesis: Proline	
89	Amino acid biosynthesis: Arginine	
90	Amino acid biosynthesis: Cysteine	2.3.1.30
91	Sulphate assimilation	
92	Amino acid biosynthesis: Cysteine	2.5.1.47
93	Amino acid biosynthesis: Histidine	
94	Amino acid biosynthesis: Isoleucine	
95	Amino acid biosynthesis: Leucine	
96	Amino acid biosynthesis: Lysine	2.7.2.4
97	Amino acid biosynthesis: Lysine	1.2.1.11
98	Amino acid biosynthesis: Lysine	4.3.3.7
99	Amino acid biosynthesis: Lysine	1.17.1.8
100	Amino acid biosynthesis: Lysine	2.6.1.83
101	Amino acid biosynthesis: Lysine	5.1.1.7
102	Amino acid biosynthesis: Lysine	4.1.1.20
103	Amino acid biosynthesis: Methionine	
104	Amino acid biosynthesis: Phenylalanine	
105	Amino acid biosynthesis: Tryptophan	4.1.3.27
106	Amino acid biosynthesis: Tryptophan	2.4.2.18
107	Amino acid biosynthesis: Tryptophan	5.3.1.24
108	Amino acid biosynthesis: Tryptophan	4.1.1.48
109	Amino acid biosynthesis: Tryptophan	4.2.1.20

110	Amino acid biosynthesis: Tyrosine	2.5.1.54
111	Amino acid biosynthesis: Tyrosine	4.2.3.4
112	Amino acid biosynthesis: Tyrosine	4.2.1.10
113	Amino acid biosynthesis: Tyrosine	1.1.1.25
114	Amino acid biosynthesis: Tyrosine	2.7.1.71
115	Amino acid biosynthesis: Tyrosine	2.5.1.19
116	Amino acid biosynthesis: Tyrosine	4.2.3.5
117	Amino acid biosynthesis: Tyrosine	5.4.99.5
118	Amino acid biosynthesis: Tyrosine	2.6.1.78
119	Amino acid biosynthesis: Tyrosine	1.3.1.78
120	Amino acid biosynthesis: Valine	
121	Nucleic acid synthesis	
122	Nucleic acid synthesis	
123	Nucleic acid synthesis	
124	Nucleic acid synthesis	
125	Nucleic acid synthesis	
126	Nucleic acid synthesis	
127	Nucleic acid synthesis	
128	Nucleic acid synthesis	
129	Synthesis of carbohydrates	
130	Synthesis of carbohydrates	
131	Synthesis of carbohydrates	
132	Synthesis of glycerophospholipids	
133	Synthesis of glycerophospholipids	
134	Synthesis of glycerophospholipids	
135	Synthesis of chlorophyll	
136	Synthesis of chlorophyll	
137	Maltose synthesis	2.7.7.27
138	Maltose synthesis	3.2.1.2
139	External exchange	
140	Synthesis of macromolecules	
141	Synthesis of macromolecules	
142	Synthesis of macromolecules	
143	Synthesis of macromolecules	

144	Synthesis of macromolecules	
145	Synthesis of chlorophyll	
146	External exchange	
147	Biomass synthesis (*C. vulgaris*)	
148	Metabolism of starch	
149	Metabolism of starch	5.4.99.16
150	Metabolism of starch	3.2.1.28
151	Glucolysis / Gluconeogenesis	
152	Glucolysis / Gluconeogenesis	2.7.1.2
153	Glucolysis / Gluconeogenesis	5.3.1.9
154	Glucolysis / Gluconeogenesis	3.1.3.11
155	Glucolysis / Gluconeogenesis	4.1.2.13
156	Glucolysis / Gluconeogenesis	5.3.1.1
157	Glucolysis / Gluconeogenesis	1.2.1.12
158	Glucolysis / Gluconeogenesis	2.7.2.3
159	Glucolysis / Gluconeogenesis	1.2.1.9
160	Glucolysis / Gluconeogenesis	5.4.2.12
161	Glucolysis / Gluconeogenesis	4.2.1.11
162	Glucolysis / Gluconeogenesis	2.7.1.40
163	Glucolysis / Gluconeogenesis	1.2.4.1
164	Glucolysis / Gluconeogenesis	1.2.4.1
165	Glucolysis / Gluconeogenesis	2.3.1.12
166	Glucolysis / Gluconeogenesis	1.8.1.4
167	Glucolysis / Gluconeogenesis	4.1.1.49
168	Glucolysis / Gluconeogenesis	6.4.1.1
169	Krebs cycle	2.3.3.1
170	Krebs cycle	4.2.1.3
171	Krebs cycle	1.1.1.42
172	Krebs cycle	1.1.1.42
173	Krebs cycle	1.2.4.2
174	Krebs cycle	1.2.4.2
175	Krebs cycle	2.3.1.61
176	Krebs cycle	6.2.1.5
177	Krebs cycle	1.3.5.1, 1.3.5.4

178	Krebs cycle	4.2.1.2
179	Krebs cycle	1.1.5.4
180	Glioxylate Cycle	4.1.3.1
181	Glioxylate Cycle	2.3.3.9
182	Glioxylate Cycle	1.1.1.38
183	Pentose Phosphate	
184	Pentose Phosphate	1.1.1.49
185	Pentose Phosphate	3.1.1.31
186	Pentose Phosphate	1.1.1.44
187	Pentose Phosphate	5.1.3.1
188	Pentose Phosphate	5.3.1.6
189	Pentose Phosphate	2.2.1.1
190	Pentose Phosphate	2.2.1.2
191	Pentose Phosphate	2.2.1.1
192	Histidine metabolism	2.7.6.1
193	Oxidative phosphorylation	1.6.1.1
194	Oxidative phosphorylation	1.6.5.3
195	Oxidative phosphorylation	1.10.3.10
196	Oxidative phosphorylation	
197	Oxidative phosphorylation	
198	Oxidative phosphorylation	3.6.3.14
199	Acetate Biosynthesis	2.7.2.1
200	Amino acid biosynthesis: Glutamate	
201	Amino acid biosynthesis: Glutamate	1.4.1.4
202	Amino acid biosynthesis: Aspartate	2.6.1.1
203	Amino acid biosynthesis: Asparagine	3.5.1.1
204	Amino acid biosynthesis: Glutamine	6.3.1.2
205	Amino acid biosynthesis: Alanine	2.6.1.2
206	Amino acid biosynthesis: Proline	1.2.1.41
207	Amino acid biosynthesis: Arginine	2.3.1.1
		2.7.2.8
		1.2.1.38
		2.6.1.11
		3.5.1.16
		6.3.5.5
		2.1.3.3
		6.3.4.5

		4.3.2.1
208	Amino acid biosynthesis: Valine, leucine and isoleucine	2.2.1.6
209	Amino acid biosynthesis: Valine, leucine and isoleucine	1.1.1.86
210	Amino acid biosynthesis: Valine, leucine and isoleucine	2.6.1.42
211	Amino acid biosynthesis: Valine, leucine and isoleucine	2.3.3.13
212	Biosynthesis of branched amino acids	4.2.1.33
213	Biosynthesis of branched amino acids	1.1.1.85
214	Amino acid biosynthesis: Valine, leucine and isoleucine	2.6.1.42
215	Amino acid biosynthesis: Valine, leucine and isoleucine	
216	Amino acid biosynthesis: Phenylalanine	
217	Amino acid biosynthesis: Phenylalanine	
218	Amino acid biosynthesis: Tyrosine	
219	Amino acid biosynthesis: Tryptophan	4.1.3.27
		2.4.2.18
		5.3.1.24
		4.2.1.20
220	Amino acid biosynthesis: Histidine	2.5.1.7
		3.5.4.19
		5.3.1.16
		2.4.2.-
		2.4.2.- 4.1.3.-
		4.2.1.19
		2.6.1.9
		1.1.1.23
221	Amino acid biosynthesis: Serine	1.1.1.95
		2.6.1.52
		3.1.3.3
222	Amino acid biosynthesis: Glycine	1.5.1.20
223	Amino acid biosynthesis: Glycine	2.1.2.1
224	Sulphate assimilation	
225	Sulphate assimilation	2.7.7.4
		2.7.1.25
		1.8.4.8
		2.1.1.107
		1.8.1.2
226	Amino acid biosynthesis: Cysteine	2.3.1.30
		2.5.1.47

		2.5.1.47
227	Amino acid biosynthesis: Homoserin	2.7.2.4
		1.2.1.11
228	Amino acid biosynthesis: Homoserin	1.1.1.3
229	Amino acid biosynthesis: Threonine	2.7.1.39
		4.2.3.1
230	Amino acid biosynthesis: Methionine	4.3.3.7
		1.17.1.8
		2.6.1.17
		2.3.1.117
		3.5.1.18
		5.1.1.7
231	Amino acid biosynthesis: Methionine	2.3.1.46
		4.4.1.11
		4.4.1.1
		2.1.1.14
		2.1.1.13
232	Amino acid biosynthesis: Lysine	4.1.1.20
233	Synthesis of ribonucleic acids	2.4.2.14
		6.3.4.13
		2.1.2.2
		6.3.3.1
		6.3.5.3
		4.1.1.21
		4.1.1.21
		6.3.4.4
		4.3.2.2
		2.1.2.3
		6.3.2.6
		2.7.4.3
234	Synthesis of ribonucleic acids	6.3.5.2
		1.1.1.205
		2.7.4.8
235	Synthesis of ribonucleic acids	2.1.3.2
		3.5.2.3
		1.3.98.1
		2.4.2.10
		4.1.1.23
		2.7.4.22
		2.7.4.6

236	Synthesis of ribonucleic acids	6.3.4.2<br>2.7.4.14
237	Synthesis of deoxyribonucleic acids	1.17.4.1<br>2.7.4.6
238	Synthesis of deoxyribonucleic acids	1.17.4.1<br>2.7.4.6
239	Synthesis of deoxyribonucleic acids	1.17.4.1<br>2.7.4.6
240	Synthesis of deoxyribonucleic acids	1.17.4.1<br>2.7.4.6
241	Synthesis of MITFS	2.3.1.41
242	Synthesis of lipopolysaccharides	5.4.2.8<br>2.7.7.9
243	Synthesis of lipopolysaccharides	
244	Synthesis of lipopolysaccharides	2.3.1.41
245	Synthesis of lipopolysaccharides	2.3.1.41
246	Synthesis of lipopolysaccharides	2.7.7.38
247	Synthesis of lipopolysaccharides	
248	Synthesis of lipopolysaccharides	
249	Synthesis of peptidoglycans	2.6.1.16
250	Synthesis of peptidoglycans	2.5.1.55
251	Synthesis of peptidoglycans	2.4.1.21
252	Synthesis of polyhydroxyalkanoates	6.4.1.2
253	Synthesis of polyhydroxyalkanoates	2.3.1.39
254	Synthesis of polyhydroxyalkanoates	2.3.1.38
255	Synthesis of polyhydroxyalkanoates	2.3.1.41
256	Synthesis of polyhydroxyalkanoates	1.1.1.100
257	Synthesis of polyhydroxyalkanoates	4.2.1.59
258	Synthesis of polyhydroxyalkanoates	1.3.1.9
259	Synthesis of polyhydroxyalkanoates	
260	Synthesis of polyhydroxyalkanoates	
261	Synthesis of polyhydroxyalkanoates	
262	Synthesis of polyhydroxyalkanoates	
263	Synthesis of polyhydroxyalkanoates	
264	Synthesis of polyhydroxyalkanoates	
265	Synthesis of polyhydroxyalkanoates	
266	Synthesis of polyhydroxyalkanoates	2.3.1.-
267	Synthesis of polyhydroxyalkanoates	

268	Synthesis of polyhydroxyalkanoates	2.3.1.-
269	Synthesis of polyhydroxyalkanoates	
270	Synthesis of polyhydroxyalkanoates	2.3.1.-
271	Synthesis of polyhydroxyalkanoates	2.3.1.-
272	Synthesis of polyhydroxyalkanoates	
273	Biomass synthesis (*P. aeruginosa*)	
274	Exchange Flows	
275	Exchange Flows	
276	Exchange Flows	
277	Exchange Flows	
278	Exchange Flows	
279	Exchange Flows	
280	Exchange Flows	
281	Exchange Flows	
282	Exchange Flows	
283	Exchange Flows	
284	Exchange Flows	
285	Exchange Flows	
286	Exchange Flows	
287	Exchange Flows	
288	Exchange Flows	
289	Exchange Flows	
290	Exchange Flows	
291	Exchange Flows	
292	Exchange Flows	
293	Exchange Flows	

Allen J (2002) Photosynthesis of ATP-electrons, proton pumps, rotors, and poise. Cell 110:273–6. doi: 10.10[illegible]
Kanehisa L (2016) KEGG: Kyoto Encyclopedia of Genes and Genomes. http://www.genome.jp/kegg/. Accessed
Nelson DL, Cox MM (2005) Lehninger Principles of Biochemistry, 4th edn. Freeman and Company, New York,
Oberhardt M a, Puchałka J, Fryer KE, et al (2008) Genome-scale metabolic network analysis of the opportunis[illegible]

Petkov G, Garcia G (2007) Which are fatty acids of the green alga Chlorella? Biochem Syst Ecol 35:281–285. d
Stephanopoulos GN, Aristidou AA, Nielsen J (1998) Metabolic Engineering: Principles and Methodologies. Ac
Yang C, Hua Q, Shimizu K (2000) Energetics and carbon metabolism during growth of microalgal cells under p

APPEND

Enzime
Photosystem I and II complex
Not apply
Chloroplast ATPase
Carbonic acid anhydrase
D-ribulose 1,5-diphosphate carboxylase
Phosphoglycerate kinase
Glyceraldehyde-3-phosphate dehydrogenase
Triose-phosphate isomerase
Fructose-bisphosphate aldolase
Fructose 1,6-bisphosphatase
Glucose-6-phosphate isomerase
Transketolase
Fructose-bisphosphate aldolase
Sedoheptulose-bisphosphatase
Transketolase
Ribulose-phosphate 3-epimerase
Ribose-5-phosphate isomerase
Phosphoribulokinase
Hexokinase
Glucose-6-phosphate isomerase
6-phosphofructokinase
Fructose 1,6-diphosphatase
Fructose-bisphosphate aldolase
Triose-phosphate isomerase
Glyceraldehyde-3-phosphate dehydrogenase
Phosphoglycerate kinase
Glyceraldehyde-3-phosphate dehydrogenase (NADP+)
2,3-diphosphoglycerate-independent phosphoglycerate mutase
Enolase
Pyruvate kinase

Pyruvate dehydrogenase (acetyl-transferring)
Pyruvate decarboxylase
Aldehyde dehydrogenase (NAD+)
Acetyl-CoA synthetase
Pyruvate carboxylase
Citrate (Si)-synthase
Aconitate hydratase
Aconitate hydratase
Isocitrate dehydrogenase (NAD+)
2-oxoglutarate dehydrogenase
2-oxoglutarate dehydrogenase
2-oxoglutarate dehydrogenase
Dihydrolipoyllysine-residue succinyltransferase
Succinyl-CoA synthetase
Succinate dehydrogenase
Fumarate hydratase
Malate dehydrogenase
Phosphoenolpyruvate carboxykinase (ATP)
D-glucose 6-phosphate dehydrogenase
6-phosphogluconolactonase
6-phosphogluconic dehydrogenase
Ribose-5-phosphate isomerase
Ribulose-phosphate 3-epimerase
Transketolase
Transketolase
Transketolase
Ribose-phosphate pyrophosphokinase
Diacylglycerol O-acyltransferase

ATP synthase
NADH:nitrate oxidoreductase, ferredoxin-nitrite reductase
Glutamate dehydrogenase (NADP+)
Glutamine synthetase
Glutamate synthase (NADPH/NADH)
Alanine transaminase
Aspartate transaminase
3-phosphoglycerate dehydrogenase
Phosphoserine aminotransferase
Phosphoserine phosphatase
Homoserine dehydrogenase
Homoserine kinase
Threonine synthase
Serine O-acetyltransferase
Cysteine synthase
Aspartate kinase
Aspartate-semialdehyde dehydrogenase
4-hydroxy-tetrahydrodipicolinate synthase
4-hydroxy-tetrahydrodipicolinate reductase
LL-diaminopimelate aminotransferase
Diaminopimelate epimerase
Diaminopimelate decarboxylase
Anthranilate synthase
Anthranilate phosphoribosyltransferase
Phosphoribosylanthranilate isomerase
Indole-3-glycerol-phosphate synthase
Tryptophan synthase

3-deoxy-7-phosphoheptulonate synthase
3-dehydroquinate synthase
3-dehydroquinate dehydratase
Shikimate dehydrogenase
Shikimate kinase
3-phosphoshikimate 1-carboxyvinyltransferase
Chorismate synthase
Chorismate mutase
Aspartate---prephenate aminotransferase
Arogenate dehydrogenase (NADP+)

glucose-1-phosphate adenylyltransferase

glycogenase

Maltose alpha-D-glucosyltransferase
Trehalase
Glucokinase
Glucose-6-phosphate isomerase
Fructose 1,6-diphosphatase
Fructose-bisphosphate aldolase
Triose-phosphate isomerase
Glyceraldehyde-3-phosphate dehydrogenase
Phosphoglycerate kinase
Glyceraldehyde-3-phosphate dehydrogenase (NADP+)
2,3-diphosphoglycerate-independent phosphoglycerate mutase
Enolase
Pyruvate kinase
Pyruvate dehydrogenase
Pyruvate dehydrogenase
Pyruvate dehydrogenase
Dihydrolipoyl dehydrogenase
Phosphoenolpyruvate carboxykinase (ATP)
Pyruvate carboxylase
Citrate (Si)-synthase
Aconitate hydratase
Iisocitrate dehydrogenase
Isocitrate dehydrogenase
2-oxoglutarate dehydrogenase
2-oxoglutarate dehydrogenase
Dihydrolipoyllysine-residue succinyltransferase
Succinyl-CoA synthetase (ADP-forming)
Succinate dehydrogenase

Fumarate hydratase
Malate dehydrogenase (quinone)
Isocitrate lyase
Malate synthase
Malate dehydrogenase
Glucose-6-phosphate-1 dehydrogenase
6-phosphogluconolactonase
Phosphogluconate dehydrogenase
Ribulose-phosphate 3-epimerase
Ribose-5-phosphate isomerase
Transketolase
Transaldolase
Transketolase
Pyrophosphoribosylphosphate synthetase
Transhydrogenase
NADH:ubiquinone reductase
Ubiquinol oxidase (H+-transporting)
H+-transporting two-sector ATPase
Acetate kinase
Alanine dehydrogenase
Aspartate transaminase
Asparaginase
Glutamine synthetase
Aminotransferase
Glutamate-5-semialdehyde dehydrogenase
Amino-acid N-acetyltransferase
Acetylglutamate kinase
N-acetyl-gamma-glutamyl-phosphate reductase
Acetylornithine transaminase
Acetylornithine deacetylase
Carbamoyl-phosphate synthase (glutamine-hydrolysing)
Ornithine carbamoyltransferase
Argininosuccinate synthase

Argininosuccinate lyase
Acetolactate synthase
Ketol-acid reductoisomerase
Branched-chain-amino-acid transaminase
2 isopropylmalate synthase
3 isopropylmalate dehydratase
3 isopropylmalate dehydrogenase
Branched-chain-amino-acid transaminase
Anthranilate synthase
Anthranilate phosphoribosyltransferase
Phosphoribosylanthranilate isomerase
Tryptophan synthase
UDP-N-acetylglucosamine 1-carboxyvinyltransferase
Phosphoribosyl-AMP cyclohydrolase
1-(5-phosphoribosyl)-5-[(5-phosphoribosylamino)methylideneamino]imidazole-4-carboxamide isomerase
Imidazole glycerol phosphate synthase, glutamine amidotransferase subunit with HisF
Glutamine amidotransferase
Imidazoleglycerol-phosphate dehydratase
Histidinol-phosphate transaminase
Histidinol dehydrogenase
Phosphoglycerate dehydrogenase
Phosphoserine transaminase
Phosphoserine phosphatase
Methylenetetrahydrofolate reductase [NAD(P)H]
Glycine hydroxymethyltransferase
Sulfate adenylyltransferase
Adenylyl-sulfate kinase
Phosphoadenosine phosphosulfate reductase
Uroporphyrinogen-III C-methyltransferase
Sulfite reductase (NADPH)
O-acetylserine synthase
Cysteine synthase A

Cysteine synthase B
Aspartate kinase
Aspartate-semialdehyde dehydrogenase
Homoserine dehydrogenase
Homoserine kinase
Threonine synthase
4-hydroxy-tetrahydrodipicolinate synthase
4-hydroxy-tetrahydrodipicolinate reductase
Succinyldiaminopimelate transaminase
2,3,4,5-tetrahydropyridine-2,6-dicarboxylate N-succinyltransferase
Succinyl-diaminopimelate desuccinylase
Diaminopimelate epimerase
homoserine O-succinyltransferase
Methionine-gamma-lyase
Cystathionine gamma-lyase
5-methyltetrahydropteroyltriglutamate--homocysteine methyltransferase
methionine synthase
Diaminopimelate decarboxylase
Amidophosphoribosyltransferase
Phosphoribosylamine---glycine ligase
Phosphoribosylglycinamide formyltransferase
Phosphoribosylformylglycinamidine cyclo-ligase
Phosphoribosylformylglycinamidine synthase
5-(carboxyamino)imidazole ribonucleotide synthase
Phosphoribosylaminoimidazole carboxylase
Adenylosuccinate synthase
Adenylosuccinate lyase
Phosphoribosylaminoimidazolecarboxamide formyltransferase / IMP cyclohydrolase
Phosphoribosylaminoimidazolesuccinocarboxamide synthase
Adenylate kinase
GMP synthase (glutamine-hydrolysing)
IMP dehydrogenase
Guanylate kinase
Aspartate carbamoyltransferase
Dihydroorotase
Dihydroorotate dehydrogenase
Orotate phosphoribosyltransferase
Orotidine-5'-phosphate decarboxylase
Uridylate kinase
Nucleoside-diphosphate kinase

Uridine triphosphate aminase
Cytidylate kinase
Ribonucleoside-diphosphate reductase beta chain
Nucleoside-diphosphate kinase
Ribonucleoside-diphosphate reductase beta chain
Nucleoside-diphosphate kinase
Ribonucleoside-diphosphate reductase beta chain
Nucleoside-diphosphate kinase
Ribonucleoside-diphosphate reductase beta chain
Nucleoside-diphosphate kinase
beta-ketoacyl-[acyl-carrier-protein] synthase I
Phosphomannomutase
UTP---glucose-1-phosphate uridylyltransferase
Ethanolamide phosphotransferase
beta-ketoacyl-[acyl-carrier-protein] synthase I
beta-ketoacyl-[acyl-carrier-protein] synthase I
3-deoxy-manno-octulosonate cytidylyltransferase
Glutamine---fructose-6-phosphate transaminase (isomerizing)
2-dehydro-3-deoxyphosphooctonate aldolase
glycogen synthase
Acetyl-CoA carboxylase
[Acyl carrier protein]malonyltransferase
[Acyl-carrier-protein] S-acetyltransferase
Beta-ketoacyl-[acyl-carrier-protein] synthase I
Beta-ketoacyl-[acyl-carrier protein](ACP) reductase
3-hydroxyacyl-[acyl-carrier-protein] dehydratase
Enoyl-[acyl carrier protein] reductase
poly(3-hydroxyalkanoic acid) synthase

poly(3-hydroxyalkanoic acid) synthase
poly(3-hydroxyalkanoic acid) synthase
poly(3-hydroxyalkanoic acid) synthase

16/S0092-8674(02)00870-X
13 Jan 2016
USA
tic pathogen Pseudomonas aeruginosa PAO1. J Bacteriol 190:2790–2803. doi: 10.1128/JE

oi: 10.1016/j.bse.2006.10.017
ademic Press, California, USA.
hotoautotrophic, mixotrophic and cyclic light-autotrophic/dark-heterotrophic conditior

IX A.- METABOLIC NETWORK OF THE CO-CULTURE *Chlorella vulgaris-Pseudomonas aeruginosa*

Biochemical reaction
9 Pho > 2 NADPH_Cv + 1 O2_Cv + 12 H_Cv
1 Piext > 1 Pi_Cv
14 H_Cv + 3 Pi_Cv > 3 ATP_Cv
1 O2_Cv > 1 O2ext
1 CO2ext > 1 CO2_Cv
1 CO2_Cv = 1 HCO3_Cv + 1 H_Cv
1 RuBP_c + 1 CO2_Cv > 2 PG3_c
1 ATP_Cv + 1 PG3_c = 1 PG13_c
1 PG13_c + 1 NADPH_Cv + 1 H_Cv > 1 G3P_c + 1 Pi_Cv
1 G3P_c > 1 G3P_Cv
1 G3P_c > 1 DAP_c
1 DAP_c + 1 G3P_c > 1 FDP_c
1 FDP_c > 1 F6P_c + 1 Pi_Cv
1 F6P_c = 1 G6P_c
1 F6P_c + 1 G3P_c > 1 E4P_c + 1 X5P_c
1 DAP_c + 1 E4P_c > 1 S17P_c
1 S17P_c > 1 S7P_c + 1 Pi_Cv
1 S7P_c + 1 G3P_c > 1 R5P_c + 1 X5P_c
1 X5P_c > 1 RL5P_c
1 R5P_c > 1 RL5P_c
1 ATP_Cv + 1 RL5P_c > 1 RuBP_c
1 GLCext > 1 GLC_Cv
1 GLC_Cv + 1 ATP_Cv > 1 G6P_Cv
1 G6P_Cv = 1 F6P_Cv
1 ATP_Cv + 1 F6P_Cv > 1 FDP_Cv
1 FDP_Cv > 1 F6P_Cv + 1 Pi_Cv
1 DAP_Cv + 1 G3P_Cv = 1 FDP_Cv
1 G3P_Cv = 1 DAP_Cv
1 G3P_Cv + 1 Pi_Cv > 1 PG13_Cv + 1 NADH_Cv
1 ATP_Cv + 1 PG3_Cv = 1 PG13_Cv
1 G3P_Cv > 1 PG3_Cv + 1 NADPH_Cv + 1 H_Cv
1 PG3_Cv = 1 PG2_Cv
1 PG2_Cv = 1 PEP_Cv
1 PEP_Cv > 1 ATP_Cv + 1 PYR_Cv

1 PYR_Cv + 1 TPP_Cv > 1 T2hPP_Cv + 1 CO2_Cv
1 T2hPP_Cv > 1 ACT_Cv + 1 TPP_Cv
1 ACT_Cv = 1 AC_Cv + 1 NADH_Cv
1 ATP_Cv + 1 AC_Cv > 1 ACCOA_Cv
1 ATP_Cv + 1 PYR_Cv + 1 HCO3_Cv > 1 Pi_Cv + 1 OA_Cv
1 ACCOA_Cv + 1 OA_Cv = 1 CIT_Cv
1 CIT_Cv = 1 ACO_Cv
1 ACO_Cv =1 ICIT_Cv
1 ICIT_Cv = 1 AKG_Cv + 1 CO2_Cv + 1 NADH_Cv + 1 H_Cv
1 AKG_Cv + 1 TPP_Cv > 1 T3hPP_Cv + 1 CO2_Cv
1 T3hPP_Cv + 1 EN6_Cv > 1 SSE_Cv + 1 TPP_Cv
1 SUCCOA_Cv + 1 EN6d_Cv = 1 SSE_Cv
1 EN6d_Cv = 1 EN6_Cv + 1 NADH_Cv + 1 H_Cv
1 Pi_Cv + 1 SUCCOA_Cv = 1 SUC_Cv + 1 ATP_Cv
1 SUC_Cv = 1 FUM_Cv
1 FUM_Cv = 1 MAL_Cv
1 MAL_Cv = 1 OA_Cv + 1 NADH_Cv + 1 H_Cv
1 ATP_Cv + 1 OA_Cv > 1 PEP_Cv + 1 CO2_Cv
1 G6P_Cv > 1 G15L6P_Cv + 1 NADPH_Cv + 1 H_Cv
1 G15L6P_Cv > 1 GL6P_Cv
1 GL6P_Cv > 1 RL5P_Cv + 1 CO2_Cv + 1 NADPH_Cv + 1 H_Cv
1 R5P_Cv = 1 RL5P_Cv
1 X5P_Cv = 1 RL5P_Cv
1 F6P_Cv + 1 G3P_Cv = 1 E4P_Cv + 1 X5P_Cv
1 S7P_Cv + 1 G3P_Cv = 1 R5P_Cv + 1 X5P_Cv
1 S7P_Cv + 1 G3P_Cv = 1 E4P_Cv + 1 F6P_Cv
1 ATP_Cv + 1 R5P_Cv = 1 PRPP_Cv
7 ACCOA_Cv + 6 ATP_Cv + 6 HCO3_Cv + 12 NADPH_Cv > 6 Pi_Cv + 6 CO2_Cv + 1 C14:0CoA
8 ACCOA_Cv + 7 ATP_Cv + 7 HCO3_Cv + 14 NADPH_Cv > 7 Pi_Cv + 7 CO2_Cv + 1 C16:0CoA
8 ACCOA_Cv + 7 ATP_Cv + 7 HCO3_Cv + 15 NADPH_Cv + 1 O2_Cv > 7 Pi_Cv + 7 CO2_Cv + 1 C16:1CoA
8 ACCOA_Cv + 7 ATP_Cv + 7 HCO3_Cv + 16 NADPH_Cv + 2 O2_Cv > 7 Pi_Cv + 7 CO2_Cv + 1 C16:2CoA
8 ACCOA_Cv + 7 ATP_Cv + 7 HCO3_Cv + 17 NADPH_Cv + 3 O2_Cv > 7 Pi_Cv + 7 CO2_Cv + 1 C16:3CoA
9 ACCOA_Cv + 8 ATP_Cv + 8 HCO3_Cv + 16 NADPH_Cv > 8 Pi_Cv + 8 CO2_Cv + 1 C18:0CoA
9 ACCOA_Cv + 8 ATP_Cv + 8 HCO3_Cv + 17 NADPH_Cv + 1 O2_Cv > 8 Pi_Cv + 8 CO2_Cv + 1 C18:1CoA
9 ACCOA_Cv + 8 ATP_Cv + 8 HCO3_Cv + 18 NADPH_Cv + 2 O2_Cv > 8 Pi_Cv + 8 CO2_Cv + 1 C18:2CoA
9 ACCOA_Cv + 8 ATP_Cv + 8 HCO3_Cv + 19 NADPH_Cv + 3 O2_Cv > 8 Pi_Cv + 8 CO2_Cv + 1 C18:3CoA
6 C14:0CoA + 250 C16:0CoA + 20 C16:1CoA + 100 C16:2CoA + 20 C16:3CoA + 4 C18:0CoA + 160 C18:1CoA + 240 C18:2CoA + 200 C18:3CoA > 1000 AcylCoA
2 NADH_Cv + 1 PG3_Cv + 3 AcylCoA >1 Pi_Cv + 1 TAG_Cv

2 NADH_Cv + 1 O2_Cv > 20 H_m
1 Pi_Cv + 3 H_m > 1 ATP_Cv
1 NO3ext + 1 NADH_Cv + 3 NADPH_Cv + 5 H_Cv > 1 NH3_Cv
1 AKG_Cv + 1 NH3_Cv + 1 NADPH_Cv + 1 H_Cv = 1 Glu_Cv
1 ATP_Cv + 1 Glu_Cv + 1 NH3_Cv > 1 Pi_Cv + 1 Gln_Cv
1 Gln_Cv + 1 AKG_Cv + 1 NADH_Cv + 1 H_Cv > 2 Glu_Cv
1 Ala_Cv + 1 AKG_Cv = 1 PYR_Cv + 1 Glu_Cv
1 Asp_Cv + 1 AKG_Cv = 1 OA_Cv + 1 Glu_Cv
1 PG3_Cv = 1 PP3_Cv + 1 NADH_Cv
1 Pser_Cv + 1 AKG_Cv = 1 PP3_Cv + 1 Glu_Cv
1 Pser_Cv > 1 Ser_Cv + 1 Pi_Cv
1 PG3_Cv + 1 NH3_Cv + 1 NADPH_Cv > 1 NADH_Cv + 1 Pi_Cv + 1 Gly_Cv
1 Hser_Cv = 1 ASPSALD_Cv + 1 NADH_Cv + 1 H_Cv
1 Hser_Cv + 1 ATP_Cv > 1 PHser_Cv
1 PHser_Cv > 1 Thr_Cv + 1 Pi_Cv
1 AKG_Cv + 1 NH3_Cv + 1 ATP_Cv + 3 NADPH_Cv > 1 Pi_Cv + 1 Prol_Cv
1 AKG_Cv + 7 ATP_Cv + 4 NADPH_Cv + 4 NH3_Cv > 1 NADH_Cv + 7 Pi_Cv + 1 Arg_Cv
1 Ser_Cv + 1 ACCOA_Cv > 1 Aser_Cv
1 SO4ext > 1 SO4_Cv
1 Aser_Cv + 1 SO4_Cv > 1 Cys_Cv + 1 AC_Cv
1 R5P_Cv + 6 ATP_Cv + 1 NADPH_Cv + 3 NH3_Cv > 6 Pi_Cv + 3 NADH_Cv + 1 His_Cv
1 OA_Cv + 1 PYR_Cv + 2 ATP_Cv + 5 NADPH_Cv + 1 NH3_Cv > 2 Pi_Cv + 1 Iso_Cv
2 PYR_Cv + 1 ACCOA_Cv + 2 NADPH_Cv + 1 NH3_Cv > 1 NADH_Cv + 1 Leu_Cv
1 ATP_Cv + 1 Asp_Cv > 1 Pasp_Cv
1 Pasp_Cv + 1 NADPH_Cv + 1 H_Cv > 1 ASPSALD_Cv + 1 Pi_Cv
1 Asp_Cv + 1 PYR_Cv > 1 sHTHP_Cv
1 sHTHP_Cv + 1 NADH_Cv + 1 H_Cv > 1 HTHP_Cv
1 HTHP_Cv + 1 Glu_Cv > 1 DIAMPIM_Cv + 1 AKG_Cv
1 DIAMPIM_Cv > 1 MDIAMPIM_Cv
1 MDIAMPIM_Cv > 1 Lys_Cv + 1 CO2_Cv
1 OA_Cv + 7 ATP_Cv + 8 NADPH_Cv + 1 NH3_Cv + 1 SO4_Cv > 7 Pi_Cv + 1 Met_Cv
2 PEP_Cv + 1 E4P_Cv + 1 ATP_Cv + 2 NADPH_Cv + 1 NH3_Cv > 1 Pi_Cv + 1 Phe_Cv
1 CHOR_Cv + 1 NH3_Cv > 1 VL1_Cv + 1 PYR_Cv
1 VL1_Cv + 1 PRPP_Cv > 1 VL15P_Cv
1 VL15P_Cv > 1 CD5P_Cv
1 CD5P_Cv > 1 I3GP_Cv + 1 CO2_Cv
1 Ser_Cv + 1 I3GP_Cv > 1 Trp_Cv + 1 G3P_Cv

1 PEP_Cv + 1 E4P_Cv > 1 DAHP_Cv + 1 Pi_Cv
1 DAHP_Cv > 1 DHQ_Cv + 1 Pi_Cv
1 DHQ_Cv > 1 DHSK_Cv
1 DHSK_Cv + 1 NADPH_Cv + 1 H_Cv > 1 Shi_Cv
1 ATP_Cv + 1 Shi_Cv > 1 Shi3P_Cv
1 PEP_Cv + 1 Shi3P_Cv > 1 Pi_Cv + 1 Shi35P_Cv
1 Shi35P_Cv > 1 CHOR_Cv + 1 Pi_Cv
1 CHOR_Cv > 1 PRE_Cv
1 PRE_Cv + 1 Asp_Cv > 1 OA_Cv + 1 ARO_Cv
1 ARO_Cv > 1 Tyr_Cv + 1 CO2_Cv + 1 NADPH_Cv + 1 H_Cv
2 PYR_Cv + 2 NADPH_Cv + 1 NH3_Cv > 1 Val_Cv
1 PG3_Cv + 1 R5P_Cv + 9 ATP_Cv + 1 NADPH_Cv + 5 NH3_Cv > 9 Pi_Cv + 3 NADH_Cv + 1 AMP_Cv
1 PG3_Cv + 1 R5P_Cv + 11 ATP_Cv + 5 NH3_Cv > 11 Pi_Cv + 3 NADH_Cv + 1 GMP_Cv
1 OA_Cv + 1 R5P_Cv + 5 ATP_Cv + 1 NADPH_Cv + 2 NH3_Cv > 5 Pi_Cv + 1 UMP_Cv
1 OA_Cv + 1 R5P_Cv + 7 ATP_Cv + 1 NADPH_Cv + 3 NH3_Cv > 5 Pi_Cv + 1 CMP_Cv
1 PG3_Cv + 1 R5P_Cv + 9 ATP_Cv + 2 NADPH_Cv + 5 NH3_Cv > 9 Pi_Cv + 3 NADH_Cv + 1 dAMP_Cv
1 PG3_Cv + 1 R5P_Cv + 11 ATP_Cv + 1 NADPH_Cv + 5 NH3_Cv > 11 Pi_Cv + 3 NADH_Cv + 1 dGMP_Cv
1 OA_Cv + 1 R5P_Cv + 5 ATP_Cv + 3 NADPH_Cv + 2 NH3_Cv > 5 Pi_Cv + 1 dTMP_Cv
1 OA_Cv + 1 R5P_Cv + 7 ATP_Cv + 2 NADPH_Cv + 3 NH3_Cv > 7 Pi_Cv + 1 dCMP_Cv
1 G6P_Cv + 1 ATP_Cv > 1 Pi_Cv + 1 UDPGLC_Cv
1 G6P_Cv + 1 ATP_Cv > 1 Pi_Cv + 1 UDPGAL_Cv
1 F6P_Cv + 1 ACCOA_Cv + 2 ATP_Cv + 1 NH3_Cv > 2 Pi_Cv + 1 UDPNAG_Cv
1 DAP_Cv + 2 AcylCoA + 1 NADH_Cv + 2 ATP_Cv + 1 Ser_Cv > 1 CO2_Cv + 1 PE_Cv
1 DAP_Cv + 1 PG3_Cv + 1 NADPH_Cv + 2 ATP_Cv + 1 NH3_Cv + 2 AcylCoA > 1 PC_Cv
2 DAP_Cv + 2 NADH_Cv + 2 AcylCoA + 2 ATP_Cv > 1 Pi_Cv + 1 PG_Cv
4 PYR_Cv + 4 G3P_Cv + 14 ATP_Cv + 29 NADPH_Cv + 8 Glu_Cv + 7 O2_Cv + 1 Mg > 10 CO2_Cv + 4 NH3_Cv + 20 Pi_Cv + 1 Chla
4 PYR_Cv + 4 G3P_Cv + 14 ATP_Cv + 31 NADPH_Cv + 8 Glu_Cv + 9 O2_Cv + 1 Mg > 10 CO2_Cv + 4 NH3_Cv + 20 Pi_Cv + 1 Chlb
10 G6P_c + 20 ATP_Cv > 3 ALMI_c
1 ALMI_c > 200 MALT_Cv
1 MALT_Cv > 1 MALText
0.79 AMP_Cv + 0.81 UMP_Cv + 0.89 GMP_Cv + 0.61 CMP_Cv + 7.44 ATP_Cv > 7.44 Pi_Cv + 1 RNA_Cv
0.79 dAMP_Cv + 0.79 dTMP_Cv + 0.82 dGMP_Cv + 0.82 dCMP_Cv + 11 ATP_Cv > 11 Pi_Cv + 1 DNA_Cv
0.612 UDPGLC_Cv + 0.102 UDPGAL_Cv + 0.286 UDPNAG_Cv > 0.578 CARBH
0.63 TAG_Cv + 0.048 PG_Cv + 0.204 PC_Cv + 0.118 PE_Cv > 0.884 LIPID

0.765 Prol_Cv + 0.126 Trp_Cv + 0.588 Asp_Cv + 0.648 Glu_Cv + 0.384 Ser_Cv + 0.298 Thr_Cv + 1.01 Gly_Cv + 1.057 Ala_Cv + 0.574 Val_Cv + 0.569 Leu_Cv + 0.326 Isc Phe_Cv + 0.189 Tyr_Cv + 0.765 Met_Cv + 0.02 Cys_Cv + 1.109 Arg_Cv + 0.26 His_Cv + 0.854 Lys_Cv + 39.1 ATP_Cv > 1.164 PRO_Cv + 39.1 Pi_Cv
2.5 Chla + 1 Chlb > 3.136 CHL
1 PO4ext > 1 PO4_Cv
95 ATP_Cv + 0.01131 K + 0.00531 Mg + 0.04214 PO4_Cv + 0.00916 SO4_Cv + 0.0027 Fe + 0.00039 Ca + 0.00003 Zn + 0.00002 Cu + 0.00006 Mn + 0.00275 DNA_Cv + 0 RNA_Cv + 0.15572 CARBH + 0.20152 LIPID + 0.53128 PRO_Cv + 0.01374 CHL > 1 BIO_Cv + 95 Pi_Cv
1 MALText > 1 MALT_P
1 MALT_P > 1 T_P
1 T_P > 2 GLC_P
1 GLCext > 1 GLC_P
1 GLC_P + 1 ATP_P > 1 G6P_P
1 G6P_P = 1 F6P_P
1 FDP_P > 1 F6P_P + 1 Pi_P
1 FDP_P = 1 G3P_P + 1 DAP_P
1 DAP_P = 1 G3P_P
1 G3P_P + 1 Pi_P = 1 PG13_P + 1 NADH_P + 1 H_P
1 ATP_P + 1 PG3_P = 1 PG13_P
1 G3P_P > 1 PG3_P + 1 NADPH_P + 1 H_P
1 PG3_P = 1 PG2_P
1 PG2_P = 1 PEP_P
1 PEP_P > 1 ATP_P + 1 PYR_P
1 PYR_P + 1 TPP_P > 1 T2hPP_P + 1 CO2_P
1 T2hPP_P + 1 EN6_P > 1 EN6s + 1 TPP_P
1 EN6s = 1 EN6_P + 1 ACCOA_P
1 EN6d_P = 1 EN6_P + 1 NADH_P + 1 H_P
1 ATP_P + 1 OA_P > 1 PEP_P + 1 CO2_P
1 ATP_P + 1 PYR_P + 1 CO2_P > 1 OA_P + 1 Pi_P
1 ACCOA_P + 1 OA_P > 1 CIT_P
1 CIT_P = 1 ICIT_P
1 ICIT_P = 1 OAS_P + 1 NADPH_P
1 OAS_P = 1 AKG_P + 1 CO2_P
1 AKG_P + 1 TPP_P > 1 T3hPP_P + 1 CO2_P
1 T3hPP_P + 1 EN6_P > 1 SSE_P + 1 TPP_P
1 SUCCOA_P + 1 EN6d_P = 1 SSE_P
1 Pi_P + 1 SUCCOA_P = 1 ATP_P + 1 SUC_P
1 SUC_P = 1 QH_P + 1 FUM_P

1 FUM_P = 1 MAL_P
1 MAL_P = 1 QH_P + 1 OA_P
1 ICIT_P > 1 GLYOx + 1 SUC_P
1 GLYOx + 1 ACCOA_P > 1 MAL_P
1 MAL_P = 1 PYR_P + 1 CO2_P + 1 NADH_P
1 CO2_P = 1 CO2ext
1 G6P_P > 1 G15L6P_P + 1 NADPH_P + 1 H_P
1 G15L6P_P > 1 GL6P_P
1 GL6P_P > 1 CO2_P + 1 NADPH_P + 1 RL5P_P
1 RL5P_P = 1 X5P_P
1 RL5P_P = 1 R5P_P
1 F6P_P + 1 G3P_P = 1 X5P_P + 1 E4P_P
1 G3P_P + 1 S7P_P = 1 F6P_P + 1 E4P_P
1 R5P_P + 1 X5P_P = 1 G3P_P + 1 S7P_P
1 ATP_P + 1 R5P_P = 1 PRPP
1 NADPH_P = 1 NADH_P
1 NADH_P > 1 QH2_P + 2 H_P
1 QH2_P + 0.5 O2_P > 2 H_P
1 O2ext > 1 O2_P
1 Piext > 1 Pi_P
1 Pi_P + 4 H_P > 1 ATP_P
1 Pi_P + 1 ACCOA_P = 1 ATP_P + 1 AC
1 NH3ext > 1 NH3_P
1 NADPH_P + 1 AKG_P + 1 NH3_P > 1 Glu_P
1 OA_P + 1 Glu_P = 1 AKG_P + 1 Asp_P
1 ATP_P + 1 Asp_P + 1 NH3_P > 1 Asn_P
1 ATP_P + 1 Glu_P + 1 NH3_P > 1 Pi_P + 1 Gln_P
1 PYR_P + 1 Glu_P = 1 AKG_P + 1 Ala_P
1 ATP_P + 2 NADPH_P + 1 Glu_P > 1 Pi_P + 1 Pro_P
4 ATP_P + 1 ACCOA_P + 1 CO2_P + 1 NADPH_P + 1 Asp_P + 2 Glu_P + 1 NH3_P > 4 Pi_P + 1 AKG_P + 1 FUM_P + 1 AC + 1 Arg_P

2 PYR_P > 1 ACLAC_P + 1 CO2_P
1 ACLAC_P = 1 OXOB_P
1 Val_P + 1 AKG_P = 1 OXOB_P + 1 Glu_P
1 OXOB_P + 1 ACCOA_P > 1 AIM_P
1 AIM_P > 1 A3IM_P
1 A3IM_P > 1 M2OX_P + 1 CO2_P + 1 NADH_P + 1 H_P
1 Leu_P + 1 AKG_P = 1 M2OX_P + 1 Glu_P
1 PYR_P + 1 NADPH_P + 1 Glu_P + 1 Thr_P > 1 CO2_P + 1 AKG_P + 1 NH3_P + 1 Iso_P
1 ATP_P + 2 PEP_P + 1 NADPH_P + 1 E4P_P > 4 Pi_P + 1 CHOR
1 Glu_P + 1 CHOR > 1 CO2_P + 1 AKG_P + 1 Phe_P
1 Glu_P + 1 CHOR > 1 CO2_P + 1 NADPH_P + 1 AKG_P + 1 Tyr_P
1 Gln_P + 1 CHOR + 1 Ser_P + 1 PRPP > 1 G3P_P + 1 PYR_P + 1 CO2_P + 1 Glu_P + 1 Trp_P
1 ATP_P + 1 Gln_P + 1 PRPP > 2 NADH_P + 1 AKG_P + 1 His_P
1 PG3_P + 1 Glu_P > 1 Pi_P + 1 NADH_P + 1 AKG_P + 1 Ser_P
1 ATP_P + 1 NADPH_P = 1 Pi_P + 1 MTHF
1 Ser_P = 1 MTHF + 1 Gly_P
1 SO4ext > 1 SO4_P
2 ATP_P + 4 NADPH_P + 1 SO4_P > 1 S_P
1 ACCOA_P + 1 Ser_P + 1 S_P > 1 AC + 1 Cys_P

1 ATP_P + 1 NADPH_P + 1 Asp_P > 1 Pi_P + 1 ASPSALD
1 NADPH_P + 1 ASPSALD > 1 Hser_P
1 ATP_P + 1 Hser_P > 1 Thr_P
1 PYR_P + 1 NADPH_P + 1 SUCCOA_P + 1 Glu_P + 1 ASPSALD > 1 AKG_P + 1 SUC_P + 1 DIAMPIM
1 SUCCOA_P + 1 MTHF + 1 Cys_P + 1 Hser_P > 1 PYR_P + 1 SUC_P + 1 NH3_P + 1 Met_P
1 DIAMPIM > 1 CO2_P + 1 Lys_P
5 ATP_P + 1 CO2_P + 1 Asp_P + 2 Gln_P + 1 PRPP + 2 MTHF + 1 Gly_P > 5 Pi_P + 1 NADPH_P + 2 FUM_P + 2 Glu_P + 1 RATP
6 ATP_P + 1 CO2_P + 1 Asp_P + 3 Gln_P + 1 PRPP + 2 MTHF + 1 Gly_P > 6 Pi_P + 1 NADH_P + 1 NADPH_P + 2 FUM_P + 3 Glu_P + 1 RGTP
4 ATP_P + 1 Asp_P + 1 NH3_P + 1 PRPP > 1 NADH_P + 1 RUTP

1 ATP_P + 1 Gln_P + 1 RUTP > 1 Pi_P + 1 Glu_P + 1 RCTP
1 NADPH_P + 1 RATP > 1 dATP
1 NADPH_P + 1 RGTP > 1 dGTP
1 NADPH_P + 1 RCTP > 1 dCTP
2 NADPH_P + 1 MTHF + 1 RUTP > 1 dTTP
7.24 ATP_P + 8.2 ACCOA_P + 13.91 NADPH_P > 7.24 Pi_P + 1 MITFS
1 ATP_P + 1 G6P_P > 1 Pi_P + 1 UDPGLC
3 ATP_P + 1 PG3_P + 1 NADPH_P + 1 NH3_P > 3 Pi_P + 1 NADH_P + 1 CDPETH
6 ATP_P + 7 ACCOA_P + 11 NADPH_P > 6 Pi_P + 1 OHMYRAC
6 ATP_P + 7 ACCOA_P + 12 NADPH_P > 6 Pi_P + 1 C14OFS
2 ATP_P + 1 PEP_P + 1 R5P_P > 2 Pi_P + 1 CMPKDO
1 ATP_P + 1.5 G6P_P > 1 Pi_P + 4 NADPH_P + 1 NDPHEP
2 ATP_P + 1 F6P_P + 1 NH3_P > 2 Pi_P + 1 TDPGLCS
1 ATP_P + 1 F6P_P + 1 ACCOA_P + 1 Gln_P > 1 Glu_P + 1 UDPNAG_P
1 PEP_P + 1 NADPH_P + 1 UDPNAG_P > 1 UDPNAM_P
1 ATP_P + 1 G6P_P > 1 Pi_P + 1 ADPGLC_P
1 ATP_P + 1 ACCOA_P + 1 CO2_P > 1 Pi_P + 1 MALCOA
1 MALCOA = 1 MALACP
1 ACCOA_P = 1 ACACP
1 MALACP + 1 ACACP > 1 ACACCOAACP
1 NADPH_P + 1 ACACCOAACP > 1 D3HBACP
1 NADPH_P + 1 D3HBACP > 1 BACP
1 NADPH_P + 1 MALACP + 1 BACP > 1 CO2_P + 1 D3HHXACP
1 NADPH_P + 1 D3HHXACP > 1 HHXACP
1 NADPH_P + 1 MALACP + 1 HHXACP > 1 CO2_P + 1 D3HOACP
1 NADPH_P + 1 D3HOACP > 1 OACP
1 NADPH_P + 1 MALACP + 1 OACP > 1 CO2_P + 1 D3HDACP
1 NADPH_P + 1 D3HDACP > 1 DACP
1 NADPH_P + 1 MALACP + 1 DACP > 1 CO2_P + 1 D3HDODACP
1 D3HOACP > 1 D3HOCOA
1 D3HOCOA > 1 PHO
1 D3HDACP > 1 D3HDCOA

1 D3HDCOA > 1 PHD
1D3HDODACP > 1 D3HDODCOA
1 D3HDODCOA > 1 PHDOD
1 PHO + 1 PHD + 1 PHDOD > 1 PHAs
1 PHAs > 1 PHAsext

23.9827 ATP_P + 0.14176 G3P_P + 0.24149 Asp_P + 0.29124 Glu_P + 0.24149 Asn_P + 0.26364 Gln_P + 0.56982 Ala_P + 0.22146 Pro_P + 0.29633 Arg_P + 0.45134 Leu_ Val_P + 0.29106 Iso_P + 0.25414 Thr_P + 0.1856 Phe_P + 0.13814 Tyr_P + 0.056944 Trp_P + 0.35794 Ser_P + 0.094911 His_P + 0.61376 Gly_P + 0.091744 Cys_P + 0.027 + 0.34378 Lys_P + 0.15397 Met_P + 0.203 RGTP + 0.136 RUTP + 0.12594 RCTP + 0.165 RATP + 0.024701 dATP + 0.025401 dGTP + 0.025401 dCTP + 0.024701 dTTP + MITFS + 0.0157 UDPGLC + 0.0235 CDPETH + 0.0235 OHMYRAC + 0.0235 C14OFS + 0.0235 CMPKDO + 0.0235 NDPHEP + 0.0157 TDPGLCS + 0.0276 UDPNAG_P + UDPNAM_P + 0.154 ADPGLC_P > 1 BIO_P + 23.98 Pi_P

> 1 Pho
> 1 Piext
> 1 K
> 1 Mg
> 1 PO4ext
> 1 SO4ext
> 1 Fe
> 1 Ca
> 1 Zn
> 1 Cu
> 1 Mn
> 1 BIO_Cv
> 1 GLCext
> 1 NO3ext
> 1 BIO_P
> 1 PHAsext
> 1 NH3ext
> 1 CO2ext
> 1 O2ext
> 1 MALText

3.01583-07

ıs. Biochem Eng J 6:87–102. doi: 10.1016/S1369-703X(00)00080-2

**Reference**
(Allen, 2002)
(Allen, 2002; Kanehisa, 2017)
(Kanehisa, 2017)
(Kanehisa, 2017)
(Kanehisa, 2017)
(Kanehisa, 2017)
(Kanehisa, 2017)
(Kanehisa, 2017)
(Kanehisa, 2017)
(Kanehisa, 2017)
(Kanehisa, 2017)
(Kanehisa, 2017)
(Kanehisa, 2017)
(Kanehisa, 2017)
(Kanehisa, 2017)
(Kanehisa, 2017)
(Kanehisa, 2017)
(Kanehisa, 2017)
(Kanehisa, 2017)
(Kanehisa, 2017)
(Kanehisa, 2017)
(Kanehisa, 2017)
(Kanehisa, 2017)
(Kanehisa, 2017)
(Kanehisa, 2017)
(Kanehisa, 2017)
(Kanehisa, 2017)
(Kanehisa, 2017)
(Kanehisa, 2017)

(Kanehisa, 2017)
(Kanehisa, 2017)
(Kanehisa, 2017)
(Kanehisa, 2017)
(Kanehisa, 2017)
(Kanehisa, 2017)
(Kanehisa, 2017)
(Kanehisa, 2017)
(Kanehisa, 2017)
(Kanehisa, 2017)
(Kanehisa, 2017)
(Kanehisa, 2017)
(Kanehisa, 2017)
(Kanehisa, 2017)
(Kanehisa, 2017)
(Kanehisa, 2017)
(Kanehisa, 2017)
(Kanehisa, 2017)
(Kanehisa, 2017)
(Kanehisa, 2017)
(Kanehisa, 2017)
(Kanehisa, 2017)
(Kanehisa, 2017)
(Kanehisa, 2017)
(Kanehisa, 2017)
(Kanehisa, 2017)
(Kanehisa, 2017)
(Petkov and Garcia, 2007)
(Kanehisa, 2017)

(Nelson and Cox, 2005)
(Kanehisa, 2017)
(Yang, et al. 2000, Kanehisa, 2017)
(Kanehisa, 2017)
(Kanehisa, 2017)
(Kanehisa, 2017)
(Kanehisa, 2017)
(Kanehisa, 2017)
(Kanehisa, 2017)
(Kanehisa, 2017)
(Kanehisa, 2017)
(Stephanopoulos *et al*.,1998)
(Kanehisa, 2017)
(Kanehisa, 2017)
(Kanehisa, 2017)
(Stephanopoulos *et al*.,1998)
(Stephanopoulos *et al*.,1998)
(Kanehisa, 2017)
(Kanehisa, 2017)
(Stephanopoulos *et al*.,1998)
(Stephanopoulos *et al*.,1998)
(Stephanopoulos *et al*.,1998)
(Kanehisa, 2017)
(Kanehisa, 2017)
(Kanehisa, 2017)
(Kanehisa, 2017)
(Kanehisa, 2017)
(Kanehisa, 2017)
(Kanehisa, 2017)
(Stephanopoulos *et al*.,1998)
(Stephanopoulos *et al*.,1998)
(Kanehisa, 2017)
(Kanehisa, 2017)
(Kanehisa, 2017)
(Kanehisa, 2017)
(Kanehisa, 2017)

(Kanehisa, 2017)
(Kanehisa, 2017)
(Kanehisa, 2017)
(Kanehisa, 2017)
(Kanehisa, 2017)
(Kanehisa, 2017)
(Kanehisa, 2017)
(Kanehisa, 2017)
(Kanehisa, 2017)
(Kanehisa, 2017)
(Stephanopoulos *et al*.,1998)
(Stephanopoulos *et al*.,1998)
(Stephanopoulos *et al*.,1998)
(Stephanopoulos *et al*.,1998)
(Stephanopoulos *et al*.,1998)
(Stephanopoulos *et al*.,1998)
(Stephanopoulos *et al*.,1998)
(Stephanopoulos *et al*.,1998)
(Stephanopoulos *et al*.,1998)
(Stephanopoulos *et al*.,1998)
(Stephanopoulos *et al*.,1998)
(Stephanopoulos *et al*.,1998)
(Stephanopoulos *et al*.,1998)
(Stephanopoulos *et al*.,1998)
(Stephanopoulos *et al*.,1998)
Sum of total reactions in Kanehisa, 2017.
Sum of total reactions in Kanehisa, 2017.
(Yang, et al. 2000)
Polysaccharides have no defined molecular mass, but are composed of at least 200 disaccharide residues (Nelson and Cox, 2005)
mm RNA of 323 g/mol (Stephanopouloset al., 1998)
mm DNA of 310 g/mol (Stephanopoulos et al., 1998)
mm de CARBH de 577.77 (Brown et al.,1997). Sialve et al., takes a value of MM of (162)n g/mol (2009)
63% mol TAG y 37% mol of total phospholipids (PC, PG, PE; Tsuzuki et al., 1990).Of the total phospholipids 32% is PE, 13% PG and 55% PC (Tsuzuki et al., 1990).MM LIPID = 884 g/mol (Sialve et al.,2009). Because the MM of phospholipids and glycerolipids is very similar, the mass% is taken as% mol.

Asn and Gln are omitted (Fowden 1951a,1951b). The value of 9.1 mmol of total amino acid is multiplied by each mole fraction of individual amino acid (Stephanopoulos et al., 1998). The value of PRO is in grams, the MM of PRO is taken as 127.89 g/mol (Fowden 1951a,b; Sialve et al. takes value from 109.5 g/mol, 2009)
Razón de 2.5 Chla / Chlb (El-Sheekh y Fathy, 2009)
For biomass composition most metabolites are obtained from Sialve et al. (2009). Total nucleic acids of 3%, (90% RNA; Janczyk et al.,2005). 1.3 % CHL, MM Chla = 892.5, MM Chlb = 906.5 (Kralovec, 2005; El-Sheekh y Fathy, 2009)
(Kanehisa, 2017)
(Oberhardt, et al. 2008)
(Kanehisa, 2017)
(Kanehisa, 2017)
(Kanehisa, 2017)
(Kanehisa, 2017)
(Kanehisa, 2017)
(Kanehisa, 2017)
(Kanehisa, 2017)
(Kanehisa, 2017)
(Kanehisa, 2017)
(Kanehisa, 2017)
(Kanehisa, 2017)
(Kanehisa, 2017)
(Kanehisa, 2017)
(Kanehisa, 2017)
(Kanehisa, 2017)
(Kanehisa, 2017)
(Kanehisa, 2017)
(Kanehisa, 2017)
(Kanehisa, 2017)
(Kanehisa, 2017)
(Kanehisa, 2017)
(Kanehisa, 2017)
(Kanehisa, 2017)
(Kanehisa, 2017)
(Kanehisa, 2017)
(Kanehisa, 2017)

(Kanehisa, 2017)
(Kanehisa, 2017)
(Kanehisa, 2017)
(Kanehisa, 2017)
(Kanehisa, 2017)
(Kanehisa, 2017)
(Kanehisa, 2017)
(Kanehisa, 2017)
(Kanehisa, 2017)
(Kanehisa, 2017)
(Kanehisa, 2017)
(Kanehisa, 2017)
(Kanehisa, 2017)
(Kanehisa, 2017)
(Kanehisa, 2017)
(Kanehisa, 2017)
(Kanehisa, 2017)
(Kanehisa, 2017)
(Kanehisa, 2017)
(Kanehisa, 2017)
(Kanehisa, 2017)
(Kanehisa, 2017)
(Kanehisa, 2017)
(Kanehisa, 2017)
(Kanehisa, 2017)
(Kanehisa, 2017)
(Kanehisa, 2017)
(Kanehisa, 2017)
(Kanehisa, 2017)
(Kanehisa, 2017)
(Kanehisa, 2017)
(Kanehisa, 2017)
(Kanehisa, 2017)

(Kanehisa, 2017)
(Kanehisa, 2017)
(Kanehisa, 2017)
(Kanehisa, 2017)
(Kanehisa, 2017)
(Kanehisa, 2017)
(Kanehisa, 2017)
(Kanehisa, 2017)
(Kanehisa, 2017)
(Kanehisa, 2017)
(Kanehisa, 2017)
(Kanehisa, 2017)
(Kanehisa, 2017)
(Kanehisa, 2017)
(Kanehisa, 2017)
(Kanehisa, 2017)
(Kanehisa, 2017)
(Kanehisa, 2017)
(Kanehisa, 2017)
(Kanehisa, 2017)
(Kanehisa, 2017)
(Kanehisa, 2017)
(Kanehisa, 2017)
(Kanehisa, 2017)
(Kanehisa, 2017)
(Kanehisa, 2017)
(Kanehisa, 2017)
(Kanehisa, 2017)
(Kanehisa, 2017)
(Kanehisa, 2017)
(Kanehisa, 2017)
(Kanehisa, 2017)

(Kanehisa, 2017)
(Kanehisa, 2017)
(Kanehisa, 2017)
(Kanehisa, 2017)
(Kanehisa, 2017)
(Kanehisa, 2017)
(Kanehisa, 2017)
(Kanehisa, 2017)
(Kanehisa, 2017)
(Kanehisa, 2017)
(Kanehisa, 2017)
(Kanehisa, 2017)
(Kanehisa, 2017)
(Kanehisa, 2017)
(Kanehisa, 2017)
(Kanehisa, 2017)
(Kanehisa, 2017)
(Kanehisa, 2017)
(Kanehisa, 2017)
(Kanehisa, 2017)
(Kanehisa, 2017)
(Kanehisa, 2017)
(Kanehisa, 2017)
(Kanehisa, 2017)
(Kanehisa, 2017)
(Kanehisa, 2017)
(Kanehisa, 2017)
(Kanehisa, 2017)
(Kanehisa, 2017)
(Kanehisa, 2017)
(Kanehisa, 2017)
(Kanehisa, 2017)
(Kanehisa, 2017)
(Kanehisa, 2017)
(Kanehisa, 2017)
(Kanehisa, 2017)
(Kanehisa, 2017)
(Kanehisa, 2017)
(Kanehisa, 2017)
(Kanehisa, 2017)

(Kanehisa, 2017)
(Kanehisa, 2017)
(Kanehisa, 2017)
(Kanehisa, 2017)
(Kanehisa, 2017)
(Kanehisa, 2017)
(Kanehisa, 2017)
(Kanehisa, 2017)
(Kanehisa, 2017)
(Kanehisa, 2017)
(Kanehisa, 2017)
(Kanehisa, 2017)
(Kanehisa, 2017)
(Kanehisa, 2017)
(Kanehisa, 2017)
(Kanehisa, 2017)
(Kanehisa, 2017)
(Kanehisa, 2017)
(Kanehisa, 2017)
(Kanehisa, 2017)
(Kanehisa, 2017)
(Kanehisa, 2017)
(Kanehisa, 2017)
(Kanehisa, 2017)
(Kanehisa, 2017)
(Kanehisa, 2017)
(Kanehisa, 2017)

(Kanehisa, 2017)
(Kanehisa, 2017)
(Kanehisa, 2017)
