# Supplementary material for: Metabolic Pathway Analysis of Nitrogen and Phosphorus Uptake by the Consortium between C. vulgaris and P. aeruginosa
Source: Int J Mol Sci. 2019 Apr 23;20(8):1978. doi: 10.3390/ijms20081978 (PMC6515159; doi:10.3390/ijms20081978)
Supplement: Supplementary file 1 [file ijms-20-01978-s001.zip › ijms-464067 supplementary/Table S2.pdf]

# APPENDIX B.- NOMENCLATURE OF COMPOUNDS

| CODE | KEGG CODE | NAME |
|---|---|---|
| A3IM_P | C04411 | (2R,3S)-3-Isopropylmalate in *P. aeruginosa* |
| AC | C00033 | Acetate in *P. aeruginosa* |
| AC_Cv | C00033 | Acetate in *C. vulgaris* |
| ACACCOAACP | C05744 | Acetoacetyl-[acyl-carrier protein] |
| ACACP | C03939 | Acetyl-[acyl-carrier protein] |
| ACCOA_Cv | C00024 | Acetyl coenzyme A in *C. vulgaris* |
| ACCOA_P | C00024 | Acetyl coenzyme A in *P. aeruginosa* |
| ACLAC_P | C06010 | (S)-2-Acetolactate in *P. aeruginosa* |
| ACO_Cv | C00417 | cis-Aconitate in *C. vulgaris* |
| ACT_Cv | C00084 | Acetaldehyde in *C. vulgaris* |
| AcylCoA | C00024 | Acetyl coenzyme A |
| ADPGLC_P | C00498 | Adenosine diphosphoglucose in *P. aeruginosa* |
| AIM_P | C02504 | alpha-Isopropylmalate in *P. aeruginosa* |
| AKG_Cv | C00026 | 2-Oxoglutarate in *C. vulgaris* |
| AKG_P | C00026 | 2-Oxoglutarate in *P. aeruginosa* |
| Ala_Cv | C00041 | L-Alanine in *C. vulgaris* |
| Ala_P | C00041 | L-Alanine in *P. aeruginosa* |
| ALMI_c | C00369 | Starch in chloroplast |
| AMP_Cv | C00020 | Adenosine 5'-monophosphate in *C. vulgaris* |
| Arg_Cv | C00062 | L-Arginine in *C. vulgaris* |
| Arg_P | C00062 | L-Arginine in *P. aeruginosa* |
| ARO_Cv | C00826 | L-Arogenate in *C. vulgaris* |
| Aser_Cv | C00979 | O-Acetyl-L-serine in *C. vulgaris* |
| Asn_P | C00152 | L-Asparagine in *P. aeruginosa* |
| Asp_Cv | C00049 | L-Aspartate in *C. vulgaris* |
| Asp_P | C00049 | L-Aspartate in *P. aeruginosa* |
| ASPSALD | C00441 | L-Aspartate 4-semialdehyde in *P. aeruginosa* |
| ASPSALD_Cv | C00441 | L-Aspartate 4-semialdehyde in *C. vulgaris* |
| ATP_Cv | C00002 | Adenosine 5'-triphosphate in *C.vulgaris* |
| ATP_P | C00002 | Adenosine 5'-triphosphate in *P. aeruginosa* |
| BACP | C05745 | Butyryl-[acyl-carrier protein] |
| BIO_Cv | | Biomass of *Chlorella vulgaris* |
| BIO_P | | Biomass of *Pseudomonas aeruginosa* |
| C14:0CoA | C02593 | Tetradecanoyl-CoA |
| C14OFS | C06424 | Tetradecanoic acid |
| C16:0CoA | C00154 | Palmitoyl-CoA |
| C16:1CoA | C08362 | Palmitoleic acid |
| C16:2CoA | C05272 | trans-2-Hexadecenoyl-CoA |

| | | |
|---|---|---|
| C16:3CoA | C05258 | (S)-3-Hydroxyhexadecanoyl-CoA |
| C18:0CoA | C00412 | Stearoyl-CoA |
| C18:1CoA | C00712 | (9Z)-Octadecenoic acid |
| C18:2CoA | C02050 | Linoleoyl-CoA |
| C18:3CoA | C16162 | alpha-Linolenoyl-CoA |
| Ca | C00076 | Calcium cation |
| CARBH | | Total carbohydrates |
| CD5P_Cv | C01302 | 1-(2-Carboxyphenylamino)-1-deoxy-D-ribulose 5-phosphate |
| CDPETH | C00570 | Cytidine diphosphate ethanolamine |
| CHL | C01793 | Chlorophyll |
| Chla | C05306 | Chlorophyll a |
| Chlb | C05307 | Chlorophyll b |
| CHOR | C00251 | Chorismate in *P. aeruginosa* |
| CHOR_Cv | C00251 | Chorismate in *C. vulgaris* |
| CIT_Cv | C00158 | Citrate in *C. vulgaris* |
| CIT_P | C00158 | Citrate in *P. aeruginosa* |
| CMP_Cv | C00055 | Cytidine-5'-monophosphate in *C. vulgaris* |
| CMPKDO | N/A | Cisteína monofosfato 2-alfa-desoxioctanoato |
| CO2_Cv | C00011 | Carbon dioxide in *C. vulgaris* |
| CO2_P | C00011 | Carbon dioxide in *P. aeruginosa* |
| CO2ext | C00011 | External carbon dioxide |
| Cu | C00070 | Copper |
| Cys_Cv | C00097 | L-Cysteine in *C. vulgaris* |
| Cys_P | C00097 | L-Cysteine in *P. aeruginosa* |
| D3HBACP | C04618 | (3R)-3-Hydroxybutanoyl-[acyl-carrier protein] |
| D3HDACP | C16220 | (R)-3-Hydroxyoctadecanoyl-[acp] |
| D3HDCOA | C05264 | (S)-Hydroxydecanoyl-CoA |
| D3HDODACP | C05757 | (R)-3-Hydroxydodecanoyl-[acp] |
| D3HDODCOA | C05262 | (S)-3-Hydroxydodecanoyl-CoA |
| D3HHXACP | C05747 | (R)-3-Hydroxyhexanoyl-[acp] |
| D3HOACP | C04620 | (3R)-3-Hydroxyoctanoyl-[acyl-carrier protein] |
| D3HOCOA | C05266 | (S)-3-Hydroxyoctanoyl-CoA |
| DACP | C05755 | Decanoyl-[acyl-carrier protein] |
| DAHP_Cv | C04691 | 2-Dehydro-3-deoxy-D-arabino-heptonate 7-phosphate in *C. vulgaris* |
| dAMP_Cv | C00360 | 2'-Deoxyadenosine 5'-phosphate in *C. vulgaris* |
| DAP_c | C00111 | Glycerone phosphate in chloroplast |
| DAP_Cv | C00111 | Glycerone phosphate in *C. vulgaris* |
| DAP_P | C00111 | Glycerone phosphate in *P. aeruginosa* |
| dATP | C00131 | 2'-Deoxyadenosine 5'-triphosphate |
| dCMP_Cv | C00239 | Deoxycytidylic acid in *C. vulgaris* |
| dCTP | C00458 | Deoxycytidine 5'-triphosphate |

| | | |
|---|---|---|
| dGMP_Cv | C00362 | 2'-Deoxyguanosine 5'-monophosphate in *C. vulgaris* |
| dGTP | C00286 | 2'-Deoxyguanosine 5'-triphosphate |
| DHQ_Cv | C00944 | 3-Dehydroquinate in *C. vulgaris* |
| DHSK_Cv | C02637 | 3-Dehydroshikimate in *C. vulgaris* |
| DIAMPIM | C00666 | LL-2,6-Diaminoheptanedioate |
| DIAMPIM_Cv | C00666 | LL-2,6-Diaminoheptanedioate in *C. vulgaris* |
| DNA_Cv | C00039 | Deoxyribonucleic acid of *C. vulgaris* |
| dTMP_Cv | C00364 | Thymidine 5'-phosphate in *C. vulgaris* |
| dTTP | C11437 | 1-Deoxy-D-xylulose 5-phosphate |
| E4P_c | C00279 | D-Erythrose 4-phosphate in chloroplast |
| E4P_Cv | C00279 | D-Erythrose 4-phosphate in *C. vulgaris* |
| E4P_P | C00279 | D-Erythrose 4-phosphate in *P. aeruginosa* |
| EN6_Cv | C15972 | Enzyme N6-(lipoyl)lysine in *C. vulgaris* |
| EN6_P | C15972 | Enzyme N6-(lipoyl)lysine in *P. aeruginosa* |
| EN6d_Cv | C15973 | Enzyme N6-(dihydrolipoyl)lysine in *C. vulgaris* |
| EN6d_P | C15973 | Enzyme N6-(dihydrolipoyl)lysine in *P. aeruginosa* |
| EN6s | C15972 | Lipoamide-E |
| F6P_c | C05345 | beta-D-Fructose 6-phosphate in chloroplast |
| F6P_Cv | C05345 | beta-D-Fructose 6-phosphate in *C. vulgaris* |
| F6P_P | C05345 | beta-D-Fructose 6-phosphate in *P. aeruginosa* |
| FDP_c | C05378 | beta-D-Fructose 1,6-bisphosphate in chloroplast |
| FDP_Cv | C05378 | beta-D-Fructose 1,6-bisphosphate in *C. vulgaris* |
| FDP_P | C05378 | beta-D-Fructose 1,6-bisphosphate in *P. aeruginosa* |
| Fe | C00023 | Iron |
| FUM_Cv | C00122 | Fumarate in *C. vulgaris* |
| FUM_P | C00122 | Fumarate in *P. aeruginosa* |
| G15L6P_Cv | C01236 | D-Glucono-1,5-lactone 6-phosphate in *C. vulgaris* |
| G15L6P_P | C01236 | D-Glucono-1,5-lactone 6-phosphate in *P. aeruginosa* |
| G3P_c | C00118 | D-Glyceraldehyde 3-phosphate in chloroplast |
| G3P_Cv | C00118 | D-Glyceraldehyde 3-phosphate in *C. vulgaris* |
| G3P_P | C00118 | D-Glyceraldehyde 3-phosphate in *P. aeruginosa* |
| G6P_c | C00668 | alpha-D-Glucose 6-phosphate in chloroplast |
| G6P_Cv | C00668 | alpha-D-Glucose 6-phosphate in *C. vulgaris* |
| G6P_P | C00668 | alpha-D-Glucose 6-phosphate in *P. aeruginosa* |
| GL6P_Cv | C00345 | 6-Phospho-D-gluconate in *C. vulgaris* |
| GL6P_P | C00345 | 6-Phospho-D-gluconate in *P. aeruginosa* |
| GLC_Cv | C00267 | alpha-D-Glucose in *C. vulgaris* |
| GLC_P | C00267 | alpha-D-Glucose in *P. aeruginosa* |
| GLCext | C00267 | External alpha-D-Glucose |
| Gln_Cv | C00064 | L-Glutamine in *C. vulgaris* |
| Gln_P | C00064 | L-Glutamine in *P. aeruginosa* |

| | | |
|---|---|---|
| Glu_Cv | C00025 | L-Glutamate in *C. vulgaris* |
| Glu_P | C00025 | L-Glutamate in *P. aeruginosa* |
| Gly_Cv | C00037 | Glycine in *C. vulgaris* |
| Gly_P | C00037 | Glycine in *P. aeruginosa* |
| GLYOx | C00048 | Glyoxylate |
| GMP_Cv | C00144 | Guanosine 5'-phosphate in *C. vulgaris* |
| H_Cv | C00080 | Hydron in *C. vulgaris* |
| H_m | C00080 | Hydron in mitochondria |
| H_P | C00080 | Hydron in *P. aeruginosa* |
| HCO3_Cv | C00288 | Bicarbonate in *C. vulgaris* |
| HHXACP | C05749 | Hexanoyl-[acyl-carrier protein] |
| His_Cv | C00135 | L-Histidine in *C. vulgaris* |
| His_P | C00135 | L-Histidine in *P. aeruginosa* |
| Hser_Cv | C00263 | L-Homoserine in *C. vulgaris* |
| Hser_P | C00263 | L-Homoserine in *P. aeruginosa* |
| HTHP_Cv | C03972 | 2,3,4,5-Tetrahydrodipicolinate in *C. vulgaris* |
| I3GP_Cv | C03506 | Indoleglycerol phosphate in *C. vulgaris* |
| ICIT_Cv | C00311 | Isocitrate in *C. vulgaris* |
| ICIT_P | C00311 | Isocitrate in *P. aeruginosa* |
| Iso_Cv | C00123 | L-Leucine in *C. vulgaris* |
| Iso_P | C00123 | L-Leucine in *P. aeruginosa* |
| K | C00238 | Potassium cation |
| Leu_Cv | C16439 | Leucine in *C. vulgaris* |
| Leu_P | C16439 | Leucine in *P. aeruginosa* |
| LIPID | | Lípidos totales |
| Lys_Cv | C00047 | L-Lysine in *C. vulgaris* |
| Lys_P | C00047 | L-Lysine in *P. aeruginosa* |
| M2OX_P | C00233 | 4-Methyl-2-oxopentanoate in *P. aeruginosa* |
| MAL_Cv | C00149 | (S)-Malate in *C. vulgaris* |
| MAL_P | C00149 | (S)-Malate in *P. aeruginosa* |
| MALACP | C01209 | Malonyl-[acyl-carrier protein] |
| MALCOA | C00083 | Malonyl coenzyme A |
| MALT_Cv | C00208 | Maltose in *C. vulgaris* |
| MALT_P | C00208 | Maltose in *P. aeruginosa* |
| MALText | C00208 | External maltose |
| MDIAMPIM_Cv | C00680 | meso-2,6-Diaminoheptanedioate |
| Met_Cv | C00073 | L-Methionine in *C. vulgaris* |
| Met_P | C00073 | L-Methionine in *P. aeruginosa* |
| Mg | C00305 | Magnesium cation |
| MITFS | | Fatty acids comprised between 16 and 18 carbon atoms |
| Mn | C00034 | Manganese |

| | | |
|---|---|---|
| MTHF | C00143 | 5,10-Methylenetetrahydrofolate |
| NADH_Cv | C00004 | Reduced nicotinamide adenine dinucleotide in *C. vulgaris* |
| NADH_P | C00004 | Reduced nicotinamide adenine dinucleotide in *P. aeruginosa* |
| NADPH_Cv | C00005 | Reduced nicotinamide adenine dinucleotide phosphate in *C. vulgaris* |
| NADPH_P | C00005 | Reduced nicotinamide adenine dinucleotide phosphate in *P. aeruginosa* |
| NDPHEP | | Mannoheptose diphosphate nucleotide |
| NH3_Cv | C00014 | Ammonia in *C. vulgaris* |
| NH3_P | C00014 | Ammonia in *P. aeruginosa* |
| NH3ext | C00014 | External ammonia |
| NO3ext | C00244 | External nitrate |
| O2_Cv | C00007 | Oxygen in *C. vulgaris* |
| O2_P | C00007 | Oxygen in *P. aeruginosa* |
| O2ext | C00007 | External oxygen |
| OA_Cv | C00036 | Oxaloacetate in *C. vulgaris* |
| OA_P | C00036 | Oxaloacetate in *P. aeruginosa* |
| OACP | C05752 | Octanoyl-[acyl-carrier protein] |
| OAS_P | C05379 | Oxalosuccinate in *P. aeruginosa* |
| OHMYRAC | C04688 | (3R)-3-Hydroxytetradecanoyl-[acyl-carrier protein] |
| OXOB_P | C00141 | 3-Methyl-2-oxobutanoic acid in *P. aeruginosa* |
| Pasp_Cv | C03082 | 4-Phospho-L-aspartate in *C. vulgaris* |
| PC_Cv | C00157 | Phosphatidylcholine in *C. vulgaris* |
| PE_Cv | C00350 | Phosphatidylethanolamine in *C. vulgaris* |
| PEP_Cv | C00074 | Phosphoenolpyruvate in *C. vulgaris* |
| PEP_P | C00074 | Phosphoenolpyruvate in *P. aeruginosa* |
| PG_Cv | C00344 | Phosphatidylglycerol in *C. vulgaris* |
| PG13_c | C00236 | 3-Phospho-D-glyceroyl phosphate in chloroplast |
| PG13_Cv | C00236 | 3-Phospho-D-glyceroyl phosphate in *C. vulgaris* |
| PG13_P | C00236 | 3-Phospho-D-glyceroyl phosphate in *P. aeruginosa* |
| PG2_Cv | C00631 | 2-Phospho-D-glycerate in *C. vulgaris* |
| PG2_P | C00631 | 2-Phospho-D-glycerate in *P. aeruginosa* |
| PG3_c | C00197 | 3-Phospho-D-glycerate in chloroplast |
| PG3_Cv | C00197 | 3-Phospho-D-glycerate in *C. vulgaris* |
| PG3_P | C00197 | 3-Phospho-D-glycerate in *P. aeruginosa* |
| PHAs | | Poly (3-hydroxyalkanoates) |
| PHAsext | | External poly (3-hydroxyalkanoates) |
| PHD | | Poly (3-hydroxydecanoate) |
| PHDOD | | Poly (3-hydroxydecanoate) |
| Phe_Cv | C00079 | L-Phenylalanine in *C. vulgaris* |
| Phe_P | C00079 | L-Phenylalanine in *P. aeruginosa* |
| Pho | | Photon |
| PHO | | Poly (3-hydroxyoctanoate) |

| | | |
|---|---|---|
| PHser_Cv | C01102 | O-Phospho-L-homoserine in *C. vulgaris* |
| Pi_Cv | C06262 | Phosphorus in *C. vulgaris* |
| Pi_P | C06262 | Phosphorus in *P. aeruginosa* |
| Piext | C06262 | External phosphorus |
| PO4_Cv | C00009 | Phosphate in *C. vulgaris* |
| PO4ext | C00009 | External phosphate |
| PP3_Cv | C03232 | 3-Phosphonooxypyruvate *in C. vulgaris* |
| PRE_Cv | C00254 | Prephenate in *C. vulgaris* |
| PRO_Cv | | Total protein in *C. vulgaris* |
| Pro_P | C00148 | L-Proline in *P. aeruginosa* |
| Prol_Cv | C00148 | L-Proline in *C. vulgaris* |
| PRPP | C00119 | 5-Phospho-alpha-D-ribose 1-diphosphate |
| PRPP_Cv | C00119 | 5-Phospho-alpha-D-ribose 1-diphosphate in *C. vulgaris* |
| Pser_Cv | C01005 | O-Phospho-L-serine in *C. vulgaris* |
| PYR_Cv | C00022 | Pyruvate in *C. vulgaris* |
| PYR_P | C00022 | Pyruvate in *P. aeruginosa* |
| QH_P | C15603 | Hydroquinone in *P. aeruginosa* |
| QH2_P | C00390 | Ubiquinol in *P. aeruginosa* |
| R5P_c | C00117 | D-Ribose 5-phosphate in chloroplast |
| R5P_Cv | C00117 | D-Ribose 5-phosphate in *C. vulgaris* |
| R5P_P | C00117 | D-Ribose 5-phosphate in *P. aeruginosa* |
| RATP | | Adenosine triphosphate ribonucleotide |
| RCTP | | Cytosine triphosphate ribonucleotide |
| RGTP | | Guanine triphosphate ribonucleotide |
| RL5P_c | C00199 | D-Ribulose 5-phosphate in chloroplast |
| RL5P_Cv | C00199 | D-Ribulose 5-phosphate in *C. vulgaris* |
| RL5P_P | C00199 | D-Ribulose 5-phosphate in *P. aeruginosa* |
| RNA_Cv | C00046 | Ribonucleic acid of *C. vulgaris* |
| RuBP_c | C01182 | D-Ribulose 1,5-bisphosphate in chloroplast |
| RUTP | | Uridine triphosphate ribonucleotide |
| S_P | C00087 | Sulfur in *P. aeruginosa* |
| S17P_c | C00447 | Sedoheptulose 1,7-bisphosphate in chloroplast |
| S7P_c | C05382 | Sedoheptulose 7-phosphate in chloroplast |
| S7P_Cv | C05382 | Sedoheptulose 7-phosphate in *C. vulgaris* |
| S7P_P | C05382 | Sedoheptulose 7-phosphate in *P. aeruginosa* |
| Ser_Cv | C00065 | L-Serine in *C. vulgaris* |
| Ser_P | C00065 | L-Serine in *P. aeruginosa* |
| Shi_Cv | C00493 | Shikimate in *C. vulgaris* |
| Shi35P_Cv | C01269 | 5-O-(1-Carboxyvinyl)-3-phosphoshikimate in *C. vulgaris* |
| Shi3P_Cv | C03175 | Shikimate 3-phosphate in *C. vulgaris* |
| sHTHP_Cv | C20258 | (2S,4S)-4-Hydroxy-2,3,4,5-tetrahydrodipicolinate in *C. vulgaris* |

| | | |
|---|---|---|
| SO4_Cv | C00059 | Sulfate in *C. vulgaris* |
| SO4_P | C00059 | Sulfate in *P. aeruginosa* |
| SO4ext | C00059 | External sulfate |
| SSE_Cv | C16255 | [Dihydrolipoyllysine-residue acetyltransferase] S-acetyldihydrolipoyllysine in *C. vulgaris* |
| SSE_P | C16255 | [Dihydrolipoyllysine-residue acetyltransferase] S-acetyldihydrolipoyllysine in *P. aeruginosa* |
| SUC_Cv | C00042 | Succinate in *C. vulgaris* |
| SUC_P | C00042 | Succinate in *P. aeruginosa* |
| SUCCOA_Cv | C00091 | Succinyl-CoA in *C. vulgaris* |
| SUCCOA_P | C00091 | Succinyl-CoA in *P. aeruginosa* |
| T_P | C01083 | Trehalose in *P. aeruginosa* |
| T2hPP_Cv | C05125 | 2-(alpha-Hydroxyethyl)thiamine diphosphate in *C. vulgaris* |
| T2hPP_P | C05125 | 2-(alpha-Hydroxyethyl)thiamine diphosphate in *P. aeruginosa* |
| T3hPP_Cv | C05381 | 3-Carboxy-1-hydroxypropyl-ThPP in *C. vulgaris* |
| T3hPP_P | C05381 | 3-Carboxy-1-hydroxypropyl-ThPP in *P. aeruginosa* |
| TAG_Cv | C00422 | Triacylglycerol in *C. vulgaris* |
| TDPGLCS | | Thymidine-5-diphosphate glucosamine |
| Thr_Cv | C00188 | L-Threonine in *C. vulgaris* |
| Thr_P | C00188 | L-Threonine in *P. aeruginosa* |
| TPP_Cv | C00068 | Thiamin diphosphate in *C. vulgaris* |
| TPP_P | C00068 | Thiamin diphosphate in *P. aeruginosa* |
| Trp_Cv | C00525 | D-Tryptophan in *C. vulgaris* |
| Trp_P | C00525 | D-Tryptophan in *P. aeruginosa* |
| Tyr_Cv | C00082 | L-Tyrosine in *C. vulgaris* |
| Tyr_P | C00082 | L-Tyrosine in *P. aeruginosa* |
| UDPGAL_Cv | C00052 | UDP-alpha-D-galactose in *C. vulgaris* |
| UDPGLC | C00029 | UDP-glucose |
| UDPGLC_Cv | C00029 | UDP-glucose in *C. vulgaris* |
| UDPNAG_Cv | C02200 | UDP-glucosamine in *C. vulgaris* |
| UDPNAG_P | C02200 | UDP-glucosamine in *P. aeruginosa* |
| UDPNAM_P | C01050 | UDP-N-acetylmuramate in *P. aeruginosa* |
| UMP_Cv | C00105 | Uridine monophosphate in *C. vulgaris* |
| Val_Cv | C00183 | L-Valine in *C. vulgaris* |
| Val_P | C00183 | L-Valine in *P. aeruginosa* |
| VL1_Cv | C00108 | Anthranilate in *C. vulgaris* |
| VL15P_Cv | C04302 | N-(5-Phospho-D-ribosyl)anthranilate in *C. vulgaris* |
| X5P_c | C00231 | D-Xylulose 5-phosphate in chloroplast |
| X5P_Cv | C00231 | D-Xylulose 5-phosphate in *C. vulgaris* |
| X5P_P | C00231 | D-Xylulose 5-phosphate in *P. aeruginosa* |
| Zn | C00038 | Zinc |
